# Cutaneous adverse reactions after COVID‐19 vaccines in a cohort of 2740 Italian subjects: An observational study

**DOI:** 10.1111/dth.15153

**Published:** 2021-10-13

**Authors:** Teresa Grieco, Patrizia Maddalena, Alvise Sernicola, Rovena Muharremi, Stefania Basili, Domenico Alvaro, Roberto Cangemi, Alfredo Rossi, Giovanni Pellacani

**Affiliations:** ^1^ Department of Clinical Internal, Anesthesiological and Cardiovascular Sciences “Sapienza” University of Rome Rome Italy; ^2^ Department of Translational and Precision Medicine “Sapienza” University of Rome Rome Italy

**Keywords:** COVID‐19 vaccine, cutaneous adverse reaction, epidemiology, incidence, SARS‐CoV‐2

## Abstract

An in‐depth characterization of the incidence, morphology, and onset of COVID‐19‐vaccines cutaneous adverse reactions is currently lacking. The existing literature on COVID‐19 vaccination‐related cutaneous adverse reactions largely focused on messenger RNA vaccines and mainly included type 1 hypersensitivity reactions, such as urticaria and angioedema. Other cutaneous manifestations are still poorly characterized and have been classified as delayed hypersensitivity rash. Our prospective observational study on a sample of 2740 subjects who underwent the COVID‐19 vaccination aimed at defining the prevalence of cutaneous adverse reactions and at identifying their timing of onset and their correlation with the administered dose. Vaccine‐related cutaneous adverse reactions occurred in 50 subjects. Patients were asked to complete a questionnaire on the type of COVID‐19 vaccine received, the time of onset of cutaneous reactions, and the dates of administration. Out of 2740 individuals who received the COVID‐19 vaccination, 50 were diagnosed with cutaneous adverse reactions to vaccine, after the first dose in 28 patients, after the second in 20, and after both in two. We reported localized injection site erythema in 12 patients and generalized cutaneous reactions in 38 patients. Our study shows that cutaneous adverse reactions to COVID‐19 vaccination are not common and most often occur after the first dose, recurring infrequently after the second dose. These reactions are usually easily manageable and, even in severe generalized cases, oral antihistamines and corticosteroids were sufficient for resolution. Therefore, except for immediate hypersensitivity reactions, cutaneous adverse reactions do not represent a contraindication to the completion of the vaccination cycle.

## 1 INTRODUCTION

Global concern for the COVID‐19 outbreak determined the urgency to develop effective vaccines. The first COVID‐19 vaccine, Comirnaty™, was granted conditional marketing approval (CMA) on December 21, 2020.^1^ Authorization for Spikevax™ (previously COVID‐19 Vaccine Moderna), for Vaxzevria™ (previously COVID‐19 Vaccine AstraZeneca) and for COVID‐19 Vaccine Janssen followed on January 6, 2021, January 29, 2021 and March 11, 2021, respectively.^2^, ^3^

Vaccination schedules involve a single dose for COVID‐19 Vaccine Janssen and two doses administered via intramuscular route for the other vaccines; the interval between doses is 21 days for Comirnaty™, 28 days for Spikevax™, and 12 weeks for Vaxzevria™. During the first phase of vaccination, priority was given to healthcare providers and elderly people.

Currently, pivotal phase 3 randomized placebo‐controlled trials provided data on the safety of the abovementioned vaccines, including reports of local injection‐site reactions, known as “COVID‐19 vaccine arm” and consisting in erythema, edema, and pain to the site of injection.^4^

The existing literature on COVID‐19 vaccination‐related cutaneous adverse reactions (CARs) has largely focused on mRNA vaccines (Comirnaty™ and Spikevax™)^5^ and mainly included type 1 hypersensitivity reactions, such as urticaria and angioedema. Other cutaneous manifestations are still poorly characterized and have been classified as delayed hypersensitivity rash.^6^, ^7^

Except for the recent American Registry systematic report of 414 CARs,^7^ a deeper characterization of the incidence, morphology, and onset of COVID‐19‐vaccines CARs is lacking.^5^, ^8^

The objective of our study was, first, to define the prevalence of CARs in our sample, second, to identify the timing of reactions and their correlation with the administered doses (first, second, or both). A third objective was to characterize the cutaneous symptoms and, finally, to report our management of COVID‐19 vaccine adverse reactions.

## 2 MATERIALS AND METHODS

From January to July 2021, we conducted a prospective observational study on a total of 2740 subjects who underwent the COVID‐19 vaccination and provided informed consent to use of their details. 2040 (74%) were health care workers and 700 (26%) were patients referring to the Department of Dermatology and Allergology of Policlinico Umberto I Hospital‐Sapienza University of Rome. 2481 (91%) of subjects received Comirnaty™, 222 (8%), Vaxzevria™, and 37 (1%) Spikevax™. No subject was administered COVID‐19 vaccine Janssen that was locally unavailable at the time of the study. Vaccine‐related CARs occurred in 50 subjects; a thorough skin examination was performed in all patients; in seven cases histology and immunohistochemistry were also carried out to better define the diagnosis. In addition, patients were asked to complete a questionnaire on the type of COVID‐19 vaccine received, the time of onset of cutaneous reactions, and the dates of administration. Comorbidities and concomitant therapies were also investigated. Serology and blood tests were carried out to investigate autoimmunity and inflammatory status.

## 3 RESULTS

Out of 2740 individuals who received the COVID‐19 vaccination during our observation period, 50 were diagnosed with CARs to vaccine, corresponding to an incidence of 31.28 cases/1000 people–year. Of these, 20 (40%) were males and 30 (60%) were females. Mean patients age was 47 (range: 22–76), and all patients were Caucasian. Thirty patients (60%) had received Comirnaty™, 16 (32%) Vaxzevria™, and 4 (8%) Spikevax™.

In 28 patients (56%) CARs followed the first dose administration; in 20 patients (40%) and two patients (4%) CARs followed the second dose administration or both doses, respectively.

Out of 28 patients manifesting CARs after the first dose, eight (29%) had received Comirnaty™, 16 (57%) Vaxzevria™, and four (14%) Spikevax™. Patients manifesting CARs either after the second dose or after both doses had received Comirnaty™ (Table 1).

**TABLE 1:** Demographic and clinical characteristics of 50 patients developing CARs, out of 2740 observed subjects that received COVID‐19 vaccination (prevalence 1.8%)

Age	22;76 years (mean = 47)
Sex	Female: 30 (60%)
	Male: 20 (40%)
Vaccine	
Comirnaty™	30 (60%)
Vaxzevria™	16 (32%)
Spikevax™	4 (8%)
Onset	
After 1st dose	28 (56%):
	Comirnaty™: 8 (16%)
	Vaxzevria™: 16 (32%)
	Spikevax™: 4 (8%)
After 2nd dose	20 (40%):
	Comirnaty™: 20 (100%)
After 1st and 2nd dose	2 (4%):
	Comirnaty™: 2 (100%)
Latency	
<7 days	34 (68%)
7–14 days	13 (26%)
>14 days	3 (6%)
Comorbidities	
Hashimoto's thyroiditis	6 (12%)
Anaphylaxis and previous ADR to vaccines	4 (8%)
Bronchial asthma	2 (4%)
Chronic kidney disease	1 (2%)
Coronaropathy	1 (2%)
Total	14 (28%)

Abbreviations: ADR, adverse drug reaction; CAR, cutaneous adverse reaction.

CARs occurred within 7 days from vaccine administration in 34 patients (68%), within 7–14 days in 13 patients (26%) and after more than 14 days in three patients (6%). They consisted of localized injection‐site erythema in 12 patients (24%) and generalized cutaneous reactions in 38 patients (76%); urticarial rashes and/or angioedema (*n* = 14; 28%), generalized pruritus (*n* = 5; 10%), toxic erythema (*n* = 4; 8%), erythema multiforme (*n* = 3; 6%), pityriasis rosea‐like eruption (*n* = 3; 6%), Stevens‐Johnson syndrome (*n* = 1; 2%), morbilliform drug exanthema (*n* = 1; 2%), lymphomatoid drug eruption resembling PLEVA (*n* = 1; 2%), erythema nodosum (*n* = 1; 2%), late onset atopic dermatitis (*n* = 1; 2%), annular lichen planus (*n* = 1; 2%), pseudo‐chilblain relapsing with necrotic features at the second dose (*n* = 1; 2%), filler injection‐site reaction (*n* = 1; 2%), and genital fixed drug eruption (*n* = 1; 2%) (Table 2).

**TABLE 2:** Characterization of cutaneous adverse reactions to COVID‐19 vaccines. The timepoints after vaccination and related vaccine doses are reported for each reaction

CARs	Frequency	Timepoint (days)	Dose
Urticarial rash/angioedema	14 (28%)	<7, 7–14	1,2
Local injection site erythema	12 (24%)	<7,7–14	1,2
Generalized pruritus	5 (10%)	7–14	2
Toxic erythema	4 (8%)	<7	2
Erythema multiforme	3 (6%)	7–14	1
Pityriasis rosea‐like eruption	3 (6%)	>14	1
Other	9 (18%)	<7, 7–14	1, 2

*Note*: Other includes Stevens‐Johnson syndrome, morbilliform drug exanthema, lymphomatoid drug reaction resembling PLEVA, erythema nodosum, late onset atopic dermatitis, annular lichen planus, pseudo‐chilblains, filler injection site reaction, and genital fixed drug eruption.

Abbreviation: CAR, cutaneous adverse reaction.

Anamnesis revealed that 14 patients (28%) had comorbidities: Hashimoto's thyroiditis (six patients), past episodes of anaphylaxis and previous adverse reactions to other vaccines (four patients), chronic kidney disease (one patient), coronaropathy (one patient), and bronchial asthma (two patients). No patients reported using anti‐inflammatory drugs after vaccine administration. Accurate history obtained from each case allowed us to rule out other plausible causes for the observed reactions. This led us to exclude a case of thoracic herpes zoster in an elderly female patient following the second dose of Comirnaty™: herpes zoster is commonly observed in the elderly and the relationship to the vaccine could not be demonstrated.

## 4 DISCUSSION

Our results showed that CARs related to COVID‐19 vaccines Comirnaty™, Spikevax™, and Vaxzevria™ are not very common, occurring in about 1.8% of individuals. A limitation of our study was that the majority of the population sample included health care workers; the incidence of CARs—especially local injection‐site reactions—may have been underestimated, due to many physicians not seeking dermatological consultation in the presence of mild cutaneous reactions. Patients manifesting CARs were mainly females (60%); however, this may be due to selection bias, since females made up 60% of our population sample. Moreover, age of our sample was not very high, as only 6.5% of subjects were aged above 65 years. CARs were chiefly caused by Comirnaty™ (60%), followed by Vaxzevria™ (32%) and Spikevax™ (8%). These percentages are influenced by the prevalence of vaccines administered in our study group; 91% of subjects, including all healthcare workers and elderly vaccinated in our hospital, received Comirnaty™, while 8% and 1% were administered Vaxzevria™ and Spikevax™, respectively. The most common generalized CARs were angioedema and urticarial rashes (28%) that in one patient presented with peculiar involvement of flexural areas and significant photosensitivity. Persistent generalized pruritus without any cutaneous manifestation (10%) and toxic erythema (8%), which is a typical drug‐related rash,^9^ were also frequent. Less common manifestations consisted in pityriasis rosea‐like eruption,^10^ erythema multiforme, Stevens‐Johnson syndrome, morbilliform drug exanthema, lymphomatoid drug reaction resembling PLEVA,^11^ erythema nodosum, annular lichen planus, genital fixed drug eruption, pseudo‐chilblain, and filler injection‐site reaction. Interestingly, one 60‐year‐old patient presented with a generalized eczematous eruption suggestive of late onset atopic dermatitis.^12^ Of note, CARs mainly followed the first dose administration (60%), with recurrences after the second dose in only 4% cases; this should encourage patients developing cutaneous reactions after the first dose to complete the vaccination cycle. Immediate hypersensitivity reactions, occurring within 4 h after first dose administration, represent a contraindication to the second dose.^13^ None of our patients developed this type of reactions, so they all completed the vaccination cycle.

In conclusion, our study shows that CARs related to COVID‐19 vaccination are not very common and most often occur after the first dose administration. Furthermore, they infrequently recur after the second dose. CARs are usually easily manageable; in severe generalized CARs oral therapy with antihistamines and low dose corticosteroids was sufficient for the resolution of manifestations. None of our patients required hospitalization. Therefore, except for immediate hypersensitivity reactions,^14^ CARs do not represent a contraindication to the completion of the vaccination cycle.^15^

## CONFLICT OF INTEREST

The authors have no competing interests to disclose.

## AUTHOR CONTRIBUTIONS

Teresa Grieco was responsible for conceptualization and writing of the original draft; Patrizia Maddalena, Alvise Sernicola, and Rovena Muharremi were responsible for investigation and writing of the original draft; Stefania Basili, Domenico Alvaro, Roberto Cangemi were responsible for investigation and conceptualization; Alfredo Rossi was responsible for investigation and supervision; Giovanni Pellacani was responsible for supervision and project administration. All authors contributed to review and editing of the manuscript.

## Data Availability

The data that support the findings of this study are available from the corresponding author upon reasonable request.
